# Dysphagia Symptoms in Patients with Postural Orthostatic Tachycardia Syndrome (POTS): A Qualitative Study

**DOI:** 10.3390/neurolint18030044

**Published:** 2026-02-25

**Authors:** Sherry Zimmermann, Svetlana Blitshteyn

**Affiliations:** 1 Department of Health Sciences, Faulkner University, Montgomery, AL 36109, USA; 2 Department of Neurology, Jacobs School of Medicine and Biomedical Sciences, University at Buffalo, Buffalo, NY 14203, USA; 3 Dysautonomia Clinic, Williamsville, NY 14221, USA

**Keywords:** dysphagia, dysautonomia, postural orthostatic tachycardia syndrome, swallowing dysfunction, qualitative study

## Abstract

**Background**: Difficulty swallowing is a common complaint in patients with postural orthostatic tachycardia syndrome (POTS), but there are no qualitative studies that examine dysphagia in patients with POTS, resulting in a significant gap in clinical understanding and research. **Methods**: A structured interview of patients with autonomic disorders was conducted utilizing the Dysphagia Handicap Index (DHI). **Results**: Eleven participants (age range 21–71, mean age 46 years, eight women) were selected using purposive sampling through online support communities and referrals from Dysautonomia Clinic. All had POTS, and eight had comorbid Ehlers–Danlos syndrome. The data gathered from participants were used to construct thematic descriptions of their lived experiences. The mean DHI score in this cohort was 4.5, indicating significant impairment in swallowing. Four themes emerged from the participant narratives: (1) the negative physical impact of dysphagia, (2) the negative psychological impact of dysphagia, (3) the impact on daily life and relationships, and (4) reduced healthcare satisfaction. **Conclusions**: We found significant impairment due to reported dysphagia symptoms in patients with POTS. Further studies are needed to elucidate the pathophysiology, severity and type of dysphagia in POTS and to develop targeted therapies.

## 1. Introduction

The autonomic nervous system (ANS) is responsible for homeostasis, blood flow and the function of all organs and body systems, including swallowing [1]. Dysautonomia occurs when the ANS does not regulate involuntary bodily functions properly or consistently, including cardiovascular, neurologic, gastrointestinal, thermoregulatory and genitourinary functions (Table 1) [2]. Common autonomic disorders include postural orthostatic tachycardia syndrome (POTS), neurocardiogenic syncope, orthostatic hypotension and autonomic dysfunction that does not qualify for specific diagnostic criteria (unspecified dysautonomia) [3].

POTS is one of the most common autonomic disorders, affecting approximately 0.1% to 1% of the United States’ population, but the incidence of POTS is at least 5 times higher due to post-COVID-19 POTS occurring as part of long COVID [3]. POTS is diagnosed based on an excessive and sustained increase in heart rate of more than 30 beats per minute (an increase of > 40 bpm in adolescents aged 12 to 19 years) within 10 min of standing or during a tilt table test in the absence of orthostatic hypotension [4]. Symptoms of orthostatic intolerance such as palpitations, lightheadedness, headache and nausea that are worse with standing and typically improve upon lying down should be present for at least 3 months [4].

Dysphagia, or impaired swallowing, has broad pathophysiologic mechanisms and etiologies and can be caused by structural or neuromuscular impairment, as well as autoimmune, oncological, and presbyphagia (age-related) causes. It negatively affects nutrition, hydration, airway safety, and quality-of-life. Swallowing function is controlled by the vagus nerve, which is responsible for swallowing coordination. Pathophysiologically, dysphagia reflects a disruption of the neural mechanisms governing swallowing, particularly involving the vagus nerve (cranial nerve X), which plays a central role in coordinating pharyngeal and laryngeal function [5,6]. The vagus nerve is also implicated in the change of heart rate during chewing and swallowing [7], confirming the neural connection between the ANS and swallowing functions.

The pharyngeal plexus, which integrates afferent and efferent pathways from the glossopharyngeal (cranial nerve IX) and vagus nerves, modulates pharyngeal contraction and proximal esophageal motility [8]. Dysfunction within these pathways can result in clinically significant swallowing impairment, as observed in neuromuscular disorders [9] and brainstem stroke [10]. These findings highlight the importance of neuroanatomical structures within the medulla and pons, where vagal motor nuclei originate, in the pathogenesis of dysphagia.

Despite the fact that the vagus nerve plays a central role in both autonomic regulation and swallowing, dysphagia has not been adequately studied as a feature of autonomic disorders outside of pure autonomic failure or multiple system atrophy (MSA) [11,12]. We aimed to understand the nature of dysphagia and its impact on physical and mental health, relationships, and experiences of healthcare in patients with POTS via a structured interview and the Dysphagia Handicap Index (DH).

## 2. Materials and Methods

### 2.1. Study Participants

Participants were recruited through online support communities and from the Dysautonomia Clinic in Williamsville, New York. Interested individuals were screened using DHI prior to their interview. The inclusion criteria were as follows: (1) Age 18 years or older. (2) POTS diagnosed by a physician using standard diagnostic criteria, which include 1. An increase in heart rate of at least 30 bpm from supine to standing position within 10 min of standing or a tilt table test 2. Absence of orthostatic hypotension 3. Symptoms of orthostatic intolerance present for at least 3 months [4]. (3) Self-reported dysphagia with a severity score of two or higher [13]. (4) Participants were required to speak English for a 45–75 min virtual, audio-recorded semi-structured interview. Individuals were excluded if dysphagia was structural or secondary to mechanical causes, such as in post-surgical changes or external compression, due to cancer, or traumatic brain injury, or if a patient had significant cognitive or communication impairments.

Participants with established esophageal disorders, including gastroesophageal reflux disease (GERD) and primary esophageal motility disorders were intentionally included to reflect the clinical heterogeneity observed in individuals with POTS. Although these conditions are often categorized as structural or primary motility abnormalities, esophageal dysphagia in this context was conceptualized as a comorbid manifestation rather than an exclusionary organic etiology. The ANS plays a critical role in regulating esophageal motility, lower esophageal sphincter tone, and peristalsis [8].

The sample size was 11 participants (9–15 participants were acceptable), consistent with research standards prioritizing thematic depth over generalizability [14,15]. This range was expected to achieve thematic saturation.

All study participants consented to participate in this study. The study was approved by the Institutional Review Board at Faulkner University’s department of Health Sciences (250916–34–1).

### 2.2. Procedures

This study employed a validated screening instrument and semi-structured interviews to collect qualitative data. The DHI was administered electronically via Google Forms to minimize participant burden and accommodate for fatigue or mobility limitations common in patients with neurologic disorders.

Participant emails were collected only with the DHI to confirm eligibility. The DHI used in this study was developed and validated by Silbergleit et al. as a patient-reported outcome measure of dysphagia-related quality-of-life [13]. The instrument consists of 25 dysphagia descriptions distributed across three subscales: physical (nine statements), functional (nine statements), and emotional impact (seven statements). Items were rated on a three-point frequency scale (never, sometimes, and always) (Supplementary Material S1 and S2).

Eligible participants completed a one-on-one semi-structured interview conducted via Microsoft Teams or an audio-recorded telephone call. Prior to each interview, informed consent was reconfirmed, and participants were reminded of their right to pause, skip questions, or withdraw at any time. Interviews followed an open-ended guide designed to elicit participants’ perceptions of the physical, emotional, and functional effects of dysautonomia-associated dysphagia. Follow up probes were used to clarify and expand responses. All interviews were audio-recorded with permission and transcribed verbatim.

Data analysis followed a structured approach which aims to understand and describe the essence of individuals’ lived experience of dysautonomia-associated dysphagia. Rather than testing hypotheses or quantifying predefined variables, phenomenological analysis seeks to explore how participants perceive, interpret, and make meaning of their experiences [16]. In clinical research, this approach is particularly valuable for elucidating symptom perception, illness experience, and the subjective impact of disease to inform patient-centered care.

Delve qualitative analysis software (www.delvetool.com) was used to support data organization and management throughout the coding process.

The study adhered to established ethical standards for research involving human participants, with consideration given to individuals with chronic illness [17]. Confidentiality was maintained by anonymizing participants using unique codes.

## 3. Results

### 3.1. Data Analysis

Data analysis was guided by two research questions:

a. How do individuals with POTS-associated dysphagia perceive their overall physical and mental health?
b. Which dysphagia symptoms are most frequently reported in this population?

Eleven participants with POTS were recruited using purposive sampling and semi-structured interviews with open-ended questions were utilized. Interviews were recorded and transcribed via Microsoft Teams (Version 1.8.00.13251). Quantitative demographic and screening data were managed using IBM SPSS Statistics (Version 30).

### 3.2. Participant Characteristics

Our cohort (*n* = 11) consisted of participants from the contiguous United States, including three males (27%) and eight females (73%) aged 21–71 years (mean age = 46 years). All participants had documented physician-diagnosed POTS (Table 2). Five participants (45%) had a formal dysphagia diagnosis confirmed by a Modified Barium Swallow study, four of whom were referred for swallowing therapy. Participants reported barriers to therapy including provider dismissal of symptoms and symptom-related fatigue.

A broad age range was included to demonstrate that a patient’s age does not appear to be a determining factor in dysautonomia-associated dysphagia.

### 3.3. Dysphagia Symptom Profiles

The mean DHI score in this cohort was 4.5, indicating significant impairment in swallowing. Participants reported heterogeneous dysphagia presentations across swallowing phases. Pharyngeal-phase impairment was reported by 75% of participants, oral-phase difficulties by 42%, bolus transfer difficulties by 75%, and esophageal dysmotility by 83%. Additionally, 41% described intermittent loss of swallowing coordination, and 33% reported fluctuating symptom severity. Dysphagia in dysautonomia was characterized by recurrent choking and globus sensation, with several participants reporting aspiration. One participant was diagnosed with aspiration pneumonia. Many described daily variability in swallowing ability which contributed to heightened vigilance and anxiety. Participants taking medications noted swallowing difficulties prior to prescription use. Physical consequences of dysphagia included unintended weight loss, nutritional deficiencies, and fatigue, which exacerbated the burden of living with POTS.

### 3.4. Themes

Four major themes emerged, reflecting the multidimensional impact of dysautonomia-associated dysphagia (Table 3):

#### 3.4.1. Negative Physical Impact of Dysphagia

Dysphagia was experienced as an unpredictable and sometimes life-threatening condition, with participants emphasizing choking risk, aspiration, and fluctuating symptom severity.

#### 3.4.2. Negative Psychological Impact of Dysphagia

Participants described significant emotional distress, including swallowing-related anxiety, embarrassment, hypervigilance during meals, and depressive symptoms. Fear of choking and social scrutiny from those that do not understand often led to avoidance behaviors.

#### 3.4.3. Impact on Daily Life and Relationships

a. Negative Social Impact

Dysphagia substantially disrupted social participation, particularly in food-centered activities. Many participants reported isolation, strained relationships, and diminished quality-of-life due to ongoing accommodations and avoidance behaviors.

b. Positive Coping Mechanisms

Despite significant challenges, participants demonstrated resilience through adaptive coping strategies that supported emotional wellbeing and social connection, including helping others with similar conditions which fostered a sense of agency and purpose.

### 3.5. Healthcare Satisfaction

#### 3.5.1. Negative Experiences

Many participants reported dismissal, fragmented care, and limited provider understanding of multisystem illness.

#### 3.5.2. Positive Provider Relationships

Some participants were able to access knowledgeable, validating practitioners and they reported improved trust and emotional wellbeing.

## 4. Discussion

This is the first qualitative study that examined the lived experiences of patients with POTS experiencing dysphagia symptoms, addressing a critical gap in the scientific literature. In this in-depth investigation, we identified that dysphagia symptoms negatively impact patients, resulting in reduced physical and mental health, with DHI scores indicating significant swallowing impairment. While dysautonomia, a broad term that encompasses any autonomic disorder or dysfunction, is recognized as a multisystem disorder with varied symptomatology [1], swallowing impairments remain underdiagnosed outside of rare autonomic disorders, such asMSA. By centering on patient narratives, this study offers novel insights into how dysphagia manifests within the context of POTS and how it adversely affects physical functioning, psychological wellbeing, social participation, and healthcare experiences. One other study investigated dysphagia in a cohort of patients with hypermobility spectrum disorder (HSD), among which 18 patients also had comorbid POTS: this subset had significantly higher dysphagia and reflux scores than patients with HSD alone [18].

The four identified themes: negative physical impact, negative psychological impact, adverse impact on daily life and relationships, and reduced healthcare satisfaction demonstrate that dysphagia is not a minor or rare symptom of POTS. In a 2015 survey of 39 POTS patients, Deb A et al. found that 41% reported swallowing difficulty [19]; however, to our knowledge, no dedicated study examining dysphagia symptoms in patients with POTS has been conducted to date. Dysphagia in POTS is a clinically significant comorbidity with profound implications for quality-of-life that represents an unmet need in patient care.

Participants reported heterogeneous manifestations of swallowing dysfunction in the oral, pharyngeal, and/or esophageal phases of swallowing, highlighting the neurological involvement characteristic of autonomic dysfunction. Borders and Troche evaluated voluntary cough effectiveness, an essential component of airway protection, in individuals with neurologic diseases [20]. Their findings highlight that dysphagia in certain nervous system disorders is associated with impaired brainstem integration of cranial nerve-mediated airway protection. In our research, symptoms such as choking, aspiration, delayed swallow initiation, globus sensation, and unintentional weight loss were common and often fluctuated in severity. This variability may be consistent with autonomic dysfunction and impaired sympathetic regulation mediated by the vagus nerve [21,22].

Several participants described the unpredictable nature of their swallowing difficulties, causing fear and anxiety around eating and drinking. This episodic presentation distinguishes POTS-associated dysphagia from dysphagia observed in stroke or progressive neurodegenerative disorders where symptom progression is often linear and predictable. Participant reports suggest that environmental triggers, physiological stressors, and general autonomic symptom burden may directly influence swallowing safety and efficacy. This finding challenges current dysphagia paradigms that assume static or progressively declining function and highlights the need for more flexible assessments and therapy guidelines. Although the vagus nerve modulates both cardiac autonomic regulation and oropharyngeal motor control, few studies concurrently assess heart rate variability, baroflex sensitivity, or laryngeal electromyography alongside swallowing function [7,23]. This gap in the literature limits the ability to identify shared vagal pathophysiology in dysphagia and dysautonomia.

Beyond physical manifestations, dysphagia resulted in a substantial psychological toll. Although studies have highlighted the role of impaired airway clearance and voluntary cough in neurodegenerative dysphagia [20], they often do not account for patient-reported outcomes, such as fear of aspiration, meal-related anxiety, and social withdrawal, thereby overlooking the critical psychosocial dimensions of dysphagia. In the present study, all participants described heightened anxiety, fear of choking, embarrassment, and constant vigilance during eating or drinking. For many, swallowing became a cognitive task rather than an automatic function. More than half of the participants reported a loss of independence, diminished social functioning, and a perceived burden on others due to dysphagia. Socially, dysphagia significantly altered participants’ relationships with others. Restaurant meals, family gatherings, and social celebrations became a source of distress. Participants described grief over the loss of spontaneity and joy associated with eating, reinforcing findings from prior dysphagia research while extending them into the context of autonomic instability. This study demonstrates that when dysphagia occurs alongside dysautonomia, psychological distress is amplified by symptom unpredictability.

Despite these challenges, several participants committed to adaptive coping strategies, including engagement in peer support communities, chronic illness advocacy, and mental health therapy with a professional who specializes in the chronic illness community. These acts fostered resilience and restored a sense of agency, drawing attention to the importance of psychosocial resources and community connection in chronic illness management.

The quality of participants’ healthcare encounters dominated their overall health perceptions. Three participants (27%) felt supported by most, but not all of their doctors. Most participants (75%) reported lengthy diagnostic delays, clinician dismissal, and a general lack of provider knowledge regarding swallowing manifestations of POTS. These experiences frequently resulted in feelings of invalidation, frustration, and mistrust in the healthcare system.

Conversely, participants who were able to access knowledgeable, validating clinicians described increased hope, trust, and satisfaction with their care. These findings indicate that provider knowledge and attitudes play a critical role in shaping patient outcomes. Fragmented care models, where symptoms are addressed in isolation rather than as part of a multisystem disorder, were perceived as particularly detrimental. Participants repeatedly emphasized the need for clinicians to understand POTS as a unified multisystemic syndrome that includes dysphagia, rather than a collection of unrelated symptoms. To this end, effective physician–patient communication and refraining from dismissing or minimizing patient complaints are essential to recognizing the multidimensional and complex nature of POTS and other autonomic disorders and to comprehensively assess and manage patients with dysautonomia [24].

The findings of this study provide empirical support for the link between POTS and dysphagia. Participants’ MBS findings and symptom descriptions align with established neuroanatomical models of swallowing control [5,8], suggesting shared physiological mechanisms across neurological conditions. Notably, this study highlights symptom variability as a defining feature of POTS-associated dysphagia. Recognition of this variability is essential for accurate diagnosis and effective treatments.

The research findings support several diagnostic and therapeutic recommendations.

### 4.1. Routine Dysphagia Screening

The literature demonstrates a high prevalence of vagal dysfunction in dysautonomia [25,26]; however, routine swallowing evaluations are not currently incorporated into standard dysautonomia diagnostic or management protocols. The inclusion of dysphagia screenings is especially crucial for patients who report choking, swallow initiation difficulties, globus sensation, or unexplained weight loss. A review by Stathopoulos and Dalakas indicated that neuropathy in Sjögren’s syndrome can involve cranial nerves IX and X, resulting in pharyngeal weakness and delayed swallowing [9]. Dysphagia in these cases is frequently attributed to xerostomia (dry mouth) or esophageal hypomotility, potentially overlooking underlying cranial nerve dysfunction. Given the prevalence and impact of swallowing difficulties reported in patients’ dysautonomia, dysphagia screenings should be considered in patients reporting dysphagia symptoms.

### 4.2. Interdisciplinary Care Models

Integrated care teams involving neurology, cardiology, gastroenterology, and speech-language pathology are critical for addressing the multisystemic nature of dysautonomia-associated dysphagia. A coordinated, autonomic model of care is reflected in the management of MSA [27,28,29] and FD [11,12]; however, this integrative framework is usually absent in patients with more prevalent forms of dysautonomia, such as POTS.

### 4.3. Recognition of Intermittent Symptoms

Clinicians should be trained to recognize the episodic nature of dysautonomia symptoms that include dysphagia. As such, instrumental assessments along with patient-reported symptom histories should be taken into account. This is relevant given the episodic nature of dysautonomia-associated dysphagia, in which fluctuations in autonomic tone could transiently impair swallowing safety. Therefore, more integrative and flexible assessment frameworks capable of capturing variability across symptoms are needed to inform targeted therapeutic strategies, including possible interventions like non-invasive vagus nerve stimulation [30].

### 4.4. Mental Health Integration

Symptoms associated with POTS may contribute to significant psychological distress and reduced health-related quality-of-life. The combined burden of autonomic instability and swallowing impairment can further exacerbate anxiety, supporting the integration of targeted mental health services within comprehensive POTS and dysphagia treatment plans. Importantly, POTS is a heterogeneous multisystemic disorder with significant neurologic, cardiovascular, gastrointestinal and other manifestations, and therefore, mental health referrals should be framed as supportive, adjunct care. As two of the participants indicated, referral to clinicians with specific expertise in chronic illness management is recommended to ensure that psychological interventions are appropriately contextualized within the patient’s underlying medical conditions.

### 4.5. Patient-Reported Outcome Measures

The scientific literature highlights the value of patient-reported symptoms in the assessment and management of autonomic disorders [31]. Wang et al. further demonstrates that incorporating patient-reported outcome (PRO) data into clinical practice can enhance symptom recognition and guide more effective management strategies [32]. The present findings reinforce the diagnostic relevance of qualitative inquiry, suggesting validated dysphagia-specific PRO measures may require adaptation for patients. For example, the DHI could be expanded to include descriptors reflecting symptom variability and altered sensorimotor perceptions (e.g., intermittent swallowing difficulty or transient loss of automaticity). Such modifications would better capture the episodic and neurogenic characteristics of dysphagia in POTS and support more accurate differential diagnosis within this population.

In summary, this qualitative study identifies dysphagia as a significant problem in a cohort of patients with POTS. By addressing the complex intersection of autonomic dysfunction and swallowing as reported by the participants’ lived experiences, this study advocates for a paradigm shift toward multidisciplinary, system-based care. Integrating these insights into clinical practice has the potential to improve diagnostic accuracy, enhance patient–healthcare practitioner relationships, and ultimately improve the quality-of-life for individuals with POTS and associated dysphagia.

### 4.6. Limitations

Several limitations warrant consideration. First, the relatively small sample size may limit the range of perspectives represented and restrict generalizability. However, qualitative research prioritizes thematic depth over statistical inference, and the sample size was sufficient to achieve thematic saturation. Second, many participants lacked objective instrumental swallowing assessments, limiting opportunities for triangulation with validated measures such as a Modified Barium Swallow test, or Fiberoptic Endoscopic Evaluation of Swallowing. This limitation reflects systemic barriers to care that are commonly reported by patients with POTS and highlights the value of research in conditions marked by diagnostic delay and variability. Third, in this study, we did not account for patients’ comorbidities, such as Ehlers–Danlos syndrome or mast cell activation syndrome, both of which can affect swallowing and other gastrointestinal symptoms. However, the presence of comorbidities is consistent with clinical experience and a large study that showed that 80% of patients with POTS have at least one or more comorbidities [33] To this end, it is unknown which diagnostic criteria were used to diagnose these comorbidities in our cohort. Finally, the cross-sectional design precluded longitudinal follow-up. Despite these limitations, the findings provide an important foundation for future interdisciplinary research integrating neuroscience with dysphagia. The goal is to improve diagnostic accuracy, clinical management, and quality-of-life for individuals with POTS and associated dysphagia symptoms.

## 5. Conclusions

In this qualitative study, we investigated the issues surrounding dysphagia symptoms in patients with POTS and their adverse impact on physical and psychological health. We found significant physical and psychological impairment in patients with POTS secondary to dysphagia symptoms. Further studies are needed to elucidate the pathophysiology, phenotypes and therapeutic modalities that can improve swallowing dysfunction in patients with common autonomic disorders.

## Figures and Tables

**Table 1:** Autonomic features in postural orthostatic tachycardia syndrome (POTS).

System	Clinical Features	Pathophysiology
Cardiovascular	Tachycardia, palpitations, syncope, lightheadedness, exercise intolerance	Impaired autonomic regulation of blood pressure and heart rate in response to postural changes, hypovolemia, preload failure
Neurologic	Migraine, cognitive symptoms (“brain fog”), dizziness, small-fiber neuropathy, sleep disturbances, fatigue	Cerebral hypoperfusion, sympathetic overactivity, parasympathetic underactivity, aberrant central autonomic networks, autoimmunity, neuroinflammation
Gastrointestinal	Acid reflux, dysphagia, bloating, nausea, gastroparesis or rapid gastric emptying, constipation, diarrhea, food sensitivities	Autonomic dysregulation results in impaired coordination of gastrointestinal tract motility, autoimmunity, mast cell hyperactivity
Thermoregulatory	Excessive sweating or inability to sweat, heat/cold intolerance, low-grade fevers	Impaired autonomic thermoregulatory control results in difficulty maintaining stable core temperature, smallfiber neuropathy, hyperadrenergic state
Genitourinary	Urinary frequency, urinary urgency, nocturia, incomplete bladder emptying, dysuria, pelvic floor dysfunction	Impaired autonomic regulation of bladder and urethral contractility and pelvic muscle tone

**Table 2:** Demographic and clinical characteristics of study participants.

Participant	Age	Sex	Dysautonomia Diagnosis	Age of Diagnosis	DHI Score	Comorbidities	Medications
1	42	Female	POTS ME/CFS	39	4	Suspected hEDS	Ivabradine Pyridostigmine
2	21	Female	POTS	19	6	vEDS	None
3	44	Male	POTS Vasovagal syncope	42	4	hEDS GERD Gastroparesis Lupus Sjögren’s MG	None
4	35	Female	POTS	35	6	EDS Dysphagia MCAS Post-exertional malaise	None
5	65	Male	POTS SFN	62	2	Spasmodic dysphonia	Baclofen Pyridostigmine Bromide Tromethamine
6	52	Male	POTS SFN	47	6	hEDS Chiari malformation GERD Arthritis	Percocet Tizanidine Lorazepam (as needed)
7	41	Female	POTS	31	5	hEDS MCAS Gastroparesis Esophageal dysmotility GERD Reynaud’s disease Primary immune deficiency Trigeminal neuralgia EBV SPS	Baclofen Cromolyn Diazepam Propranolol Topiramate Tagamet Vonoprazan Montelukast Cetirizine Rosuvastatin Levothyroxine Carbamazepine Albuterol (as needed) SCIG
8	57	Female	POTS SFN	54	4	hEDS Sjögren’s syndrome Endometriosis PVCs and PACs Trigeminal neuralgia MCAS, Esophageal dysmotility GERD	Cyclosporine eye drops Pindolol Cevimaline Aformoterol Loteprednol Budesonide Dextro- amphetamine
9	36	Female	POTS SFN	35	4	hEDS MCAS GERD MCTD Dysphagia	Pyridostigmine
10	44	Female	POTS	40	4	Esophageal dysmotility Gastroparesis GERD Reynaud’s disease May–Thurner syndrome Achalasia Dysphagia	None
11	71	Female	POTS	69	5	Rheumatoid arthritis Gastroparesis	None

**Table 3:** Themes identified in patients with POTS reporting dysphagia symptoms and experiences.

Themes	Descriptions	Representative Codes	Example Quotations
Negative Physical Impact of Dysphagia	The physical symptoms and health consequences associated with dysphagia, including choking, difficulty swallowing, and related health risks.	Physical distress, aspiration risk	“My throat is closing up and I can’t swallow and I’m choking.”
			“I choke on saliva.”
			“I can’t get my tongue and throat to work together and then I can’t clear it (food) out of my throat.”
		Inconsistent swallowing	“Like I could be fine today and not fine tomorrow.”
			“It (swallowing problem) is baffling; it comes out of nowhere.”
		Nutritional deficiency	“My hair is falling out a lot. I can see patches of scalp.”
			“I am malnourished. My muscle mass is low and I have no energy.”
			“I’m weaker. I can’t do as much; it wipes you out.”
Negative Psychological Impact of Dysphagia	The emotional effects of dysphagia, including anxiety, feelings of isolation, and decreased self-esteem.	Emotional burden, anxiety	“It’s brutal being terrified to swallow.”
			“Every single time I swallow, I’m scared.”
			“I can’t be independent.”
		Feelings of depression	“Depression that just is right there, waiting to suck you in.”
			“I wish I were dead.” ^a^
Impact on Daily Life and Relationships	The effects of dysphagia on social interactions, relationships, and daily routines, including both negative social impacts and positive coping mechanisms.	Social withdrawal	“I accommodate all the time, all day, every day.”
			“I don’t go to events.”
			“I have lost contact with several friends.”
		Positive community engagement	“I’m going to find the people that are struggling and help them.”
Healthcare Satisfaction	The level of satisfaction with healthcare experiences, encompassing both negative experiences with providers and positive relationships that facilitate care.	Provider dismissal	“I’d like to find someone who could give some answers.”
			“Providers need to understand the umbrella of a syndrome; that it requires holistic (not fragmented) management of symptoms.”
			“I was accused of attention-seeking.”
			“Pain and suffering need to be validated.”
		Supportive provider relationships	“I have actually a very good EDS doctor who I feel supported by.”

^a^ Not actual suicidal ideation. Participant confirmed that it is extremely frustrating living with chronic pain and dysphagia, especially with family financial responsibilities.

## Data Availability

The data presented in this study are available on request from the corresponding author.

## Supplementary Materials

The following supporting information can be downloaded at https://www.mdpi.com/article/10.3390/neurolint18030044/s1, Supplementary Material S1: Quality-of-life questionnaire regarding eating and drinking; Supplementary Material S2: Semi-structured interview questions [13].

## Abbreviations

EBV	Epstein–Barr Virus
FD	Familial Dysautonomia
FEES	Fiberoptic Endoscopic Evaluation of Swallowing
GERD	Gastroesophageal Reflux Disease
hEDS	Hypermobile Ehlers–Danlos Syndrome
MBS	Modified Barium Swallow
MCTD	Mixed Connective Tissue Disease
ME/CFS	Myalgic Encephalomyelitis/Chronic Fatigue Syndrome
MG	Myasthenia Gravis
MSA	Multiple System Atrophy
OH	Orthostatic Hypotension
POTS	Postural Orthostatic Tachycardia Syndrome
SFN	Small-Fiber Neuropathy
SPS	Stiff Person Syndrome
vEDS	Vascular Ehlers–Danlos Syndrome
