# Supplementary material for: Dysphagia Symptoms in Patients with Postural Orthostatic Tachycardia Syndrome (POTS): A Qualitative Study
Source: Neurol Int. 2026 Feb 25;18(3):44. doi: 10.3390/neurolint18030044 (PMC13029299; doi:10.3390/neurolint18030044)
Supplement: Supplementary file 1 [file neurolint-18-00044-s001.zip › neurolint-4134432-supplementary.docx]

**Supplemental material S1:**

**Dysphagia Handicap Index (DHI)**

**Quality of Life Questionnaire Regarding Your Eating and Drinking**

***Chart created from the Dysphagia Handicap Index***

We are interested in learning as much as possible about your swallowing and eating abilities. Please take a minute to complete the questionnaire below. All responses will remain confidential.

**Please circle the descriptions that best describe your current diet**:

*Liquids*: Regular liquid Nectar thick Honey thick No liquids

*Solids*: Regular food Dysphagia Advanced Ground Puree (soft foods, meats cut small) (moist-easy to chew) (blended)

**Please place a check in the box that describes your swallowing difficulty**.

|  | Code |  | **Never** | **Sometimes** | **Always** |
| --- | --- | --- | --- | --- | --- |
| 1. | (1P) | I cough when I drink liquids. |  |  |  |
| 2. | (2P) | I cough when I eat solid food. |  |  |  |
| 3. | (3P) | My mouth is dry. |  |  |  |
| 4. | (4P) | I need to drink fluids to wash food down. |  |  |  |
| 5. | (5P) | I’ve lost weight because of my  swallowing problem. |  |  |  |
| 6. | (1F) | I avoid some foods because of my swallowing problem. |  |  |  |
| 7. | (2F) | I have changed the way I swallow to make it easier to eat. |  |  |  |
| 8. | (1E) | (1E) I’m embarrassed to eat in public. |  |  |  |
| 9. | (3F) | It takes me longer to eat a meal than it used to. |  |  |  |
| 10. | (4F) | I eat smaller meals more often due to my swallowing problem. |  |  |  |
| 11. | (6P) | I have to swallow again before food will go down. |  |  |  |
| 12. | (2E) | (2E) I feel depressed because I can’t eat what I want. |  |  |  |
| 13 | (3E) | (3E) I don’t enjoy eating as much as I used to. |  |  |  |

|  |  |  | **Never** | **Sometimes** | **Always** |
| --- | --- | --- | --- | --- | --- |
| 14. | (5F) | I don’t socialize as much due to my swallowing problem. |  |  |  |
| 15. | (6F) | I avoid eating because of my  swallowing problem. |  |  |  |
| 16. | (7F) | I eat less because of my swallowing problem. |  |  |  |
| 17. | (4E) | I am nervous because of my  swallowing problem. |  |  |  |
| 18. | (5E) | I feel handicapped because of my swallowing problem. |  |  |  |
| 19. | (6E) | I get angry at myself because of my swallowing problem. |  |  |  |
| 20. | (7P) | I choke when I take my medication. |  |  |  |
| 21. | (7E) | I’m afraid that I’ll choke and stop breathing because of my swallowing problem. |  |  |  |
| 22. | (8F) | I must eat another way (e.g., feeding tube) because of my swallowing problem. |  |  |  |
| 23. | (9F) | I’ve changed my diet due to my swallowing problem. |  |  |  |
| 24. | (8P) | I feel a strangling sensation when I swallow. |  |  |  |
| 25. | (9P) | I cough up food after I swallow. |  |  |  |

**Please circle the number that matches the overall severity of your swallowing difficulty**

(1 = no difficulty at all; 4 = somewhat of a problem; 7 = the worse problem you could have)

_________________________________________________________________ 1 2 3 4 5 6 7 Normal Moderate Problem Severe Problem _________________________________________________________________

Reference:

Silbergliet, A., Schultz, L., Jacobson, B., Beardsley, T., and Johnson, A. (2012). The dysphagia handicap index: Development and validation. *Dysphagia, 27*, 46-52.

**Supplemental material S2:
Semi-Structured Interview Questions**

*Background and Lived Experience*

1. What is your age?
2. Please tell me about your journey with dysautonomia.

- What type of dysautonomia have you been diagnosed with by your doctor: POTS? vasovagal syncope? orthostatic hypotension? autonomic neuropathy?
- How long have you had it for?
- Tell me about the symptoms that have had the most impact.

1. Have you been diagnosed by a physician with the following comorbidities (disorders related to dysautonomia):

- hypermobile-Ehlers-Danlos syndrome (h-EDS) or hypermobility spectrum disorder (HSD)
- mast cell activation syndrome (MCAS)
- small fiber neuropathy (SFN)
- esophageal dysmotility
- eosinophilic esophagitis
- gastroparesis
- gastroesophageal disease (GERD)
- autoimmune disease like Sjogren's, rheumatoid arthritis, lupus or celiac disease

1. How long have you had difficulty swallowing?
2. Tell me about a typical day of eating and drinking for you.

*Impact on Health and Well-Being*

1. How has dysphagia affected your physical health?

- Have you lost weight?
  - How much weight have you lost and what was the timeframe?

1. Have you noted any difficulty maintaining your nutrition?

- Have you had vitamin/mineral deficiencies on a blood test?
- Have you had changes in appearance that could indicate nutritional deficiencies like dry skin, brittle hair, ridged or curved nails?
- Have you had cognitive or mood changes?
- Do you feel like you’re able to drink enough during the day?
- Are there any foods you avoid because they are too difficult to chew or swallow?
- Have you had any recent lung infections/pneumonia?

1. Tell me about your overall satisfaction with your current health status.

- Has this changed over time?

*Psychosocial and Emotional Impact*

1. How has living with dysautonomia and dysphagia affected your mental or emotional health?
2. Have these conditions impacted your social life or relationships?

- Social eating, attending events, isolation

1. Have these conditions created financial stress?
2. Have you experienced anxiety regarding eating and drinking?
3. Has dealing with your swallowing difficulties been time consuming?

- Thickening fluids, specialized cooking

1. Do you feel understood and supported by people in your life?

- Family, friends, healthcare providers

*Healthcare Access and Satisfaction*

1. Please describe your experiences with healthcare providers regarding dysphagia.

- Have they been knowledgeable and/or helpful?
- Has a healthcare professional ever recommended a swallowing evaluation? (modified barium swallow, FEES, upper endoscopy)
- If so, what were the results?
- Have you received a referral to an SLP for swallowing therapy or diet modifications?
- Have you received a referral to a dietitian?

1. Have you been able to access therapy for your swallowing difficulties?

- If yes, what tests were completed?
- Did the SLP recommend diet modifications or exercises?
- How many therapy sessions did you receive?

1. What has helped you most in managing your dysphagia symptoms?
2. What has not helped with managing your dysphagia symptoms?

*Reflections*

1. If you could change one thing about the way your dysphagia or dysautonomia has been managed, what would it be?
2. What do you wish others better understood about your experiences?

- Family, friends, healthcare providers

1. Is there anything else you would like to share about how these conditions have affected your satisfaction with your overall health?
